# Magnolol Suppresses Pancreatic Cancer Development *In Vivo* and *In Vitro via* Negatively Regulating TGF-β/Smad Signaling

**DOI:** 10.3389/fonc.2020.597672

**Published:** 2020-12-02

**Authors:** Shuo Chen, Jiaqi Shen, Jing Zhao, Jiazhong Wang, Tao Shan, Junhui Li, Meng Xu, Xi Chen, Yang Liu, Gang Cao

**Affiliations:** ^1^ Department of General Surgery, The Second Affiliated Hospital of Xi’an Jiaotong University, Xi’an Jiaotong University, Xi'an, China; ^2^ School of Life Science, Xiamen University, Xiamen, China; ^3^ School of Science, Xi’an Jiaotong University, Xi'an, China

**Keywords:** magnolol, pancreatic cancer, TGF-β, epithelial-mesenchymal-transition, Smad

## Abstract

Magnolol, a hydroxylated biphenyl extracted from *Magnolia officinalis*, has recently drawn attention due to its anticancer potential. The present study was aimed to explore the effects of Magnolol on restraining the proliferation, migration and invasion of pancreatic cancer *in vivo* and *in vitro*. Magnolol showed significant anti-growth effect in an orthotopic xenograft nude mouse model, and immunohistochemical staining of the xenografts revealed that Magnolol suppressed vimentin expression and facilitated E-cadherin expression. The cytoactive detection using CCK-8 assay showed Magnolol inhibited PANC-1 and AsPC-1 concentration-dependently. Scratch healing assay and the Transwell invasion assay proved the inhibiting effects of Magnolol on cellular migration and invasion at a non-cytotoxic concentration. Western blot and rt-PCR showed that Magnolol suppressed epithelial-mesenchymal-transition by increasing the expression level of E-cadherin and decreasing those of N-cadherin and vimentin. Magnolol suppressed the TGF-β/Smad pathway by negatively regulating phosphorylation of Smad2/3. Moreover, TGF-β1 impaired the antitumor effects of Magnolol *in vivo*. These results demonstrated that Magnolol can inhibit proliferation, migration and invasion *in vivo* and *in vitro* by suppressing the TGF-β signal pathway and EMT. Magnolol could be a hopeful therapeutic drug for pancreatic malignancy.

## Introduction

Pancreatic cancer is a common malignancy and ranks as the 7th leading cancer-associated mortality in developed nations (1). Although great improvement has been reached in surgery, chemotherapeutics and radiotherapeutics, the prognosis is still poor due to the local invasion and metastasis upon diagnosis (2, 3). Furthermore, no definite cure strategy can be provided to treat the patients with advanced stages or metastasis (2). Therefore, it is urgent to develop new and effective drugs to treat pancreatic cancer.

Epithelial-mesenchymal-transition (EMT) is an important segment in cancer invasion, metastasis, proliferation and maintenance of stem cell characteristics (4). When EMT occurs, tumor cells lose epithelial integrity and cell polarity and gain an invasive and motile phenotype (5). Accumulated evidence demonstrates that transforming growth factor-β (TGFβ) signaling is a prime inducer of EMT in the tumor microenvironment (6). The increased pretreatment of soluble TGF‐β1 in serum of unresectable pancreatic cancer patients indicates a poor prognosis for chemotherapy (7).

Magnolol (MAG, C_18_H_18_O_2_, CAS Number 528-43-8, PubChem CID 72300) is a natural hydroxylated biphenyl extracted from the root and stem bark of *Magnolia officinalis*, *Magnolia obovata* and *Magnolia grandiflora* (8). Magnolol is reported to have a good safety profile and anti-tumor effects in accumulated studies. Magnolol mediates cell death *via* PI3K/Akt-mediated epigenetic modifications and benefits the management of melanoma (9). Magnolol inhibits the growth and invasion of cholangiocarcinoma cells *via* the NF-kappaB pathway (10). However, whether Magnolol inhibits the EMT and invasion and migration *in vivo*, how it restrains growth of the tumor *in vitro* remains unclear.

In the present study, a pancreatic orthotopic xenograft in a model nude mouse was applied to access the effect of Magnolol on the tumor growth, and pancreatic cancer cell lines (Panc-1 and AsPC-1) were employed to evaluate the impacts of Magnolol on cell biological behavior. TGF-β1-induced EMT was also investigated as a potential mechanism of action of Magnolol.

## Materials and Methods

### Chemicals

Magnolol (C_18_H_18_O_2_, 5’-Diallyl-2,2’-dihydroxybiphenyl, molecular weight: 266.33, purity ≥98%) was purchased from Aladdin Company (Cat. M111378, Aladdin, Shanghai, China). Dimethyl sulfoxide (DMSO, Aladdin, Shanghai, China) was used as solvent. A stock solution of magnolol (125 mM, 332.9 mg magnolol in 10 ml DMSO) was stored at −15°C. Magnolol stock was diluted in PBS (1.5mM for magnolol, 1.2% v/v for DMSO) and PBS-DMSO (1.2%) was served as a negative control. Recombinant Human Transforming Growth Factor β1 (TGF-β1) was obtained from R&D systems (Cat. 240-B-002, Shanghai, China) and dissolved with sterile ddH_2_O when need.

### Cell Culture

The human pancreatic cancer cell lines PANC-1 and AsPC-1 were obtained from American Type Culture Collection (ATCC, Manassas, VA, USA) and were cultured with DMEM medium with 10% fetal bovine serum (Gibco), 100 U/ml penicillin, and 100 μg/ml streptomycin in incubators under 5% CO_2_ at 37°C.

### CCK-8 Assay

Approximately 2,000 cells were inoculated in each well of 96-well plates and incubated with a concentration range of Magnolol. A Cell Counting Kit-8 (ab228554, abcam, shanghai China) was applied to test the cellular viability. The experiments were performed five times independently.

### Colony Formation Assay

Approximately 200 cells were seeded in each well of 6-well culture plates, and the plates were incubated for 10 h. Then, fresh media containing 0, 15, 30 μM Magnolol were applied to replace the old media, and the samples were then cultured for 14 days. The colonies were fixed in 4% paraformaldehyde (Aladdin, Shanghai, China) and then stained with crystal violet (Aladdin, Shanghai, China). Formatted colonies were gently washed and air seasoned before photos of them were taken. The experiments were performed three times independently.

### Transwell Assay for Invasion

Transwell chambers with 24 wells and 8.0-μm pore membranes (Corning USA) were applied following the manufacturer’s protocol. Approximately 100,000 cells were seeded in each well in the upper chamber. The upper chamber was filled with serum-free medium which was covered by thin layers of matrigel basement membrane matrix while the lower chamber was filled with complete medium. The invaded cells were fixed with 4% paraformaldehyde and stained with 0.1% crystal violet solution after being incubated for 24 h at 37°C. Photos were taken using light microscopy and the invaded cells in five random fields were counted.

### Scratch-Wound Migration Assay

Cells were seeded and confluently cultured in 6-well plates. The scratches were made by scraping with a sterile 1-ml pipette tip in confluently cultured cells. The medium was changed to medium containing 0, 15, or 30 μM Magnolol, and the samples were incubated for 48 h. Photos were captured using a phase-contrast microscope at 0, 24, and 48 h. The rate of cell migration equals the ratio of the coalesced area of the scratch in 48 h to total the area of the scratch at 0 h. The experiments were performed three times independently.

### Evaluation of Cell Morphology

To measure changes in cell shape during EMT, we applied the published methods (11). Photos taken by an inverted phase contrast microscope during cell culture were analyzed by Image Plus software. Cell area, major axis, minor axis of random 50 cells per condition were collected and used to calculate the hybrid morphology parameters. Roundness (4 × area/(π × major axis)^2^), for which a high value indicates a high degree of roundness in cell shape (11). Aspect ratio (major axis/minor axis), for which a high value indicates an elongated cell shape (11).

### Western Blot

RIPA buffer (Beyotime, China) was applied to obtain total protein from cells. Same amounts of protein were isolated by SDS-PAGE and then passed on to a polyvinylidene difluoride membrane (Millipore). The membrane was incubated with primary antibodies against E-cadherin (wl01482, WanleiBio, Shenyang, China), vimentin (WL01960, WanleiBio), pSMAD2/3 (WL02305, WanleiBio), SMAD2/3 (WL01520, WanleiBio), and β-actin (WL10372, WanleiBio) and were then incubated with HPR-conjugated secondary antibody. Chemiluminescence reagents (WanleiBio) were applied to visualize Protein bands. The experiments were performed three times independently.

### Orthotopic Xenograft Study

Five to six-week-old BALB/C nude mice were purchased from Shanghai SLAC Animal Center (Shanghai, China). An orthotopic xenograft nude mouse model was established by Orthotopic Injection Technique as described previously (12). Briefly, luciferase-tagged AsPC-1 cells (1×10^6^/50 μl) were injected into the pancreas of immunocompromised mice. Pinholes were sutured with Prolene 7-0. Once orthotopic xenograft became palpable (approximately 7 days after injection), the mice were divided into two groups randomly (four mice per group). The MAG group received i.p. injection of Magnolol (50 mg/kg, once daily), and the Control group received vehicle (equal amount of DMSO) only. Tumor growth was assessed per week by bioluminescence imaging following intravenous d-luciferin injection (150 mg/kg). Final imaging was obtained at 28 days, and then mice were sacrificed. The primary orthotopic xenografts were harvested, weighed, and fixed in Bouin’s solution.

### Immunohistochemical Staining

Paraffin specimens of harvested xenografts were cut into 4-μm-thick sections and mounted on silanized slides. The immunohistochemical staining was performed as published (13). The antibodies used were anti-Ki67 (Abcam, ab15580), anti-E-cadherin (Abcam, ab231303), anti-vimentin (Abcam, ab92547) and goat anti-rabbit secondary antibody (Abcam, ab205719). Finally, 3,3-diaminobenzidine was applied to visualize the results, and photographs were taken under a microscope (×100 and ×400).

### Statistical Analysis

All data are expressed as the mean ± SD and were analyzed using Student’s t test or one-way ANOVA where necessary. GraphPad Prism V6 was applied to analyze and visualize the data. All experiments were performed independently at least 3 times. A P value more than 0.05 was defined as statistically significant.

## Results

### MAG Inhibits Pancreatic Orthotopic Xenograft Growth in a Mouse Model

**Figure 1:**
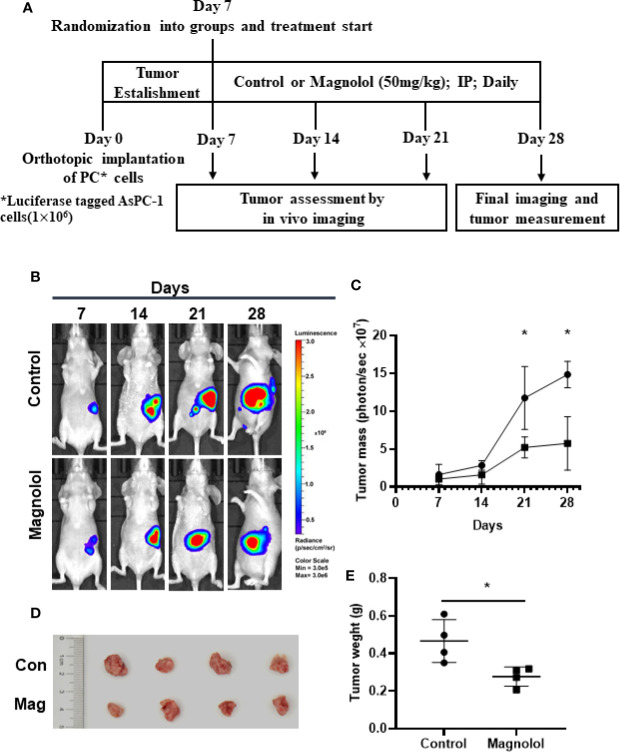
Magnolol (MAG) inhibits pancreatic orthotopic xenograft growth in a mouse model. **(A)** Schematic representation of *in vivo* treatment strategy. **(B)** Images by the living imaging analysis of mice from both the groups at different time points. **(C)** Tumor growth curve (total photons per second) showing tumor growth at different time points. **(D)** The image and **(E)** weight of the tumors harvested at the end point. *P < 0.05.

We assessed the antitumor effect of MAG with a pancreatic orthotopic xenograft in a mouse model. We chose AsPC-1 for its high tumorigenicity in mice. The experimental design is shown in **Figure 1A**. The living imaging analysis revealed that xenograft progression with MAG treatment was significantly slower compared with that in the control group (**Figures 1B, C**). Additionally, the final harvested xenografts had an average weight of 0.46 g in MAG group compared with 0.28 g in the control group (**Figures 1D, E**). There were no differences among groups besides tumor size, while no signs of toxicity.

### EMT and Proliferation Is Restrained in Pancreatic Xenografts of MAG-Treated Mice

**Figure 2:**
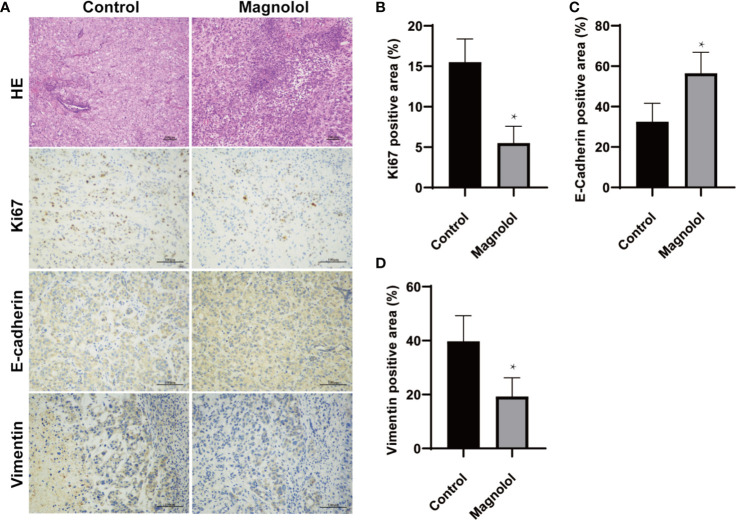
Epithelial-mesenchymal-transition (EMT) and proliferation is restrained in pancreatic xenografts of Magnolol (MAG)-treated mice. **(A)** HE staining was applied to confirm the xenografts formation while Immunohistochemical staining was used to access the expression of Ki67, E-cadherin and vimentin. **(B–D)** The expression of Ki67, E-cadherin and vimentin were analyzed. *P < 0.05.

EMT plays a critical role in cancer proliferation and maintenance of stem cell characteristics, which is reported to be significant from experimental and clinical points of view (4). We further assessed the effect of MAG on the EMT and proliferation in harvested xenografts. The proliferation and EMT was assessed using immunohistochemical staining for Ki67, E-cadherin and vimentin which are widely accepted markers (14–16). The results showed intense staining of Ki67 and vimentin in xenograft sections from the control group, whereas weak staining was detected in the MAG group (**Figures 2A, B, D**). Furthermore, strong staining of E-cadherin was detected in xenograft sections from the MAG group, while weak staining was detected in the control group (**Figures 2A, C**). These results indicate that MAG restrains EMT and proliferation in pancreatic xenografts in mice.

### MAG Suppressed the Viability of Pancreatic Cancer Cells

**Figure 3:**
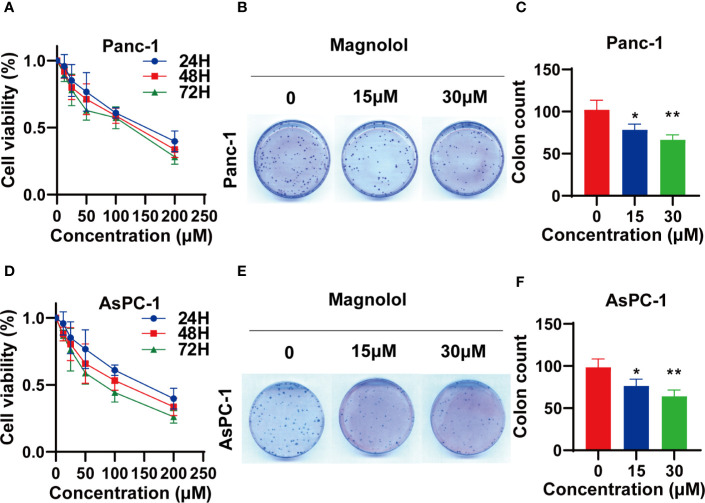
Magnolol (MAG) suppressed the viability of pancreatic cancer cells **(A, D)** MAG suppressed the viability of Panc-1 and AsPC-1 in time- and concentration-dependent manner. **(B, C, E, F)** MAG (15 or 30 μM) reduced colony formation of Panc-1 and AsPC-1. *P < 0.05; **P < 0.01.

The impact of Magnolol on the viability of PANC-1 and AsPC-1 cells was determined using an MTT assay. PANC-1 and AsPC-1 cells were incubated with a concentration range of Magnolol for 24, 48, and 72 h. The data revealed that Magnolol suppressed the viability of these cells both time- and concentration-dependently (**Figures 3A, D**). Respectively, the half inhibition concentrations (IC50) on Panc-1 cells were 140.5, 117.3, and 96.4 μM for 24, 48, and 72 h, while the IC50 values on the AsPC-1 cells were 160.0, 104.2, and 75.4 μM. The IC20 and IC10 of Magnolol of Panc-1 cell were 30.7 and 14.0 μM for 48 h, while those of the AsPC-1 cells were 24.6 and 10.6 μM. Panc-1 and AsPC-1 cells incubated with Magnolol at 15 and 30 μM for 14 days showed compromised capability in colony formation assay (**Figures 3B, C, E, F**).

### Magnolol Suppressed Cellular Migration and Invasion *In Vitro*

**Figure 4:**
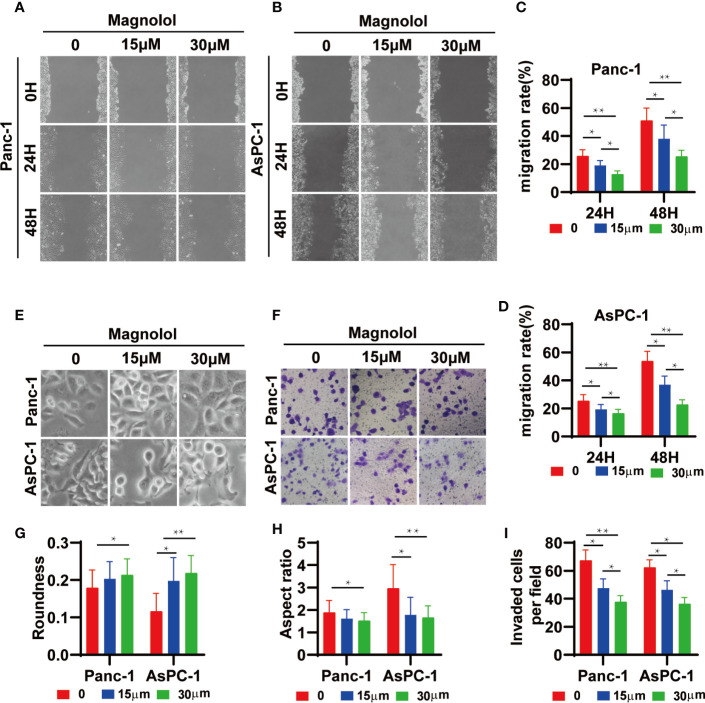
Magnolol Suppressed Cellular Migration and Invasion *in vitro* **(A, B)** The wound-healing of cells treated with Magnolol(0, 15, 30 μM) was shown at 0, 24 and 48 h after scratching. **(C, D)** The results of the scratch-wound assay were analyzed. **(E)** Morphological changes in Panc-1 and AsPC-1 cells after culture with Magnolol(0, 15, 30 μM). **(G, H)** Evaluation of cell morphology for Panc-1 and AsPC-1 cells after culture with Magnolol(0, 15, 30 μM). **(F, I)** Transwell invasion assay showed Magnolol treatment reduced the invasive rate of PANC-1 and AsPC-1 cells. *P < 0.05; **P < 0.01.

The scratch-wound assay showed that Magnolol treatment weakened the migration of cancer cells (**Figures 4A–D**). MAG-treated cells displayed epithelial-like phenotype while untreated cells showed mesenchymal-like phenotype in morphology (**Figure 4E**). The treatment of Magnolol (30 μM) in PANC-1 and (15μM & 30 μM) in AsPC-1 caused significant increase in Roundness and decrease in Aspect ratio (**Figures 4G, H**). Magnolol treatment reduced the invasive rate of PANC-1 and AsPC-1 cells in transwell invasion assay (**Figures 4F, I**). These findings indicated that the inhibiting effects of Magnolol on migration and invasion may be related to EMT.

### Magnolol Inhibited EMT *via* the Suppression of TGF-β1/Smad Signaling

**Figure 5:**
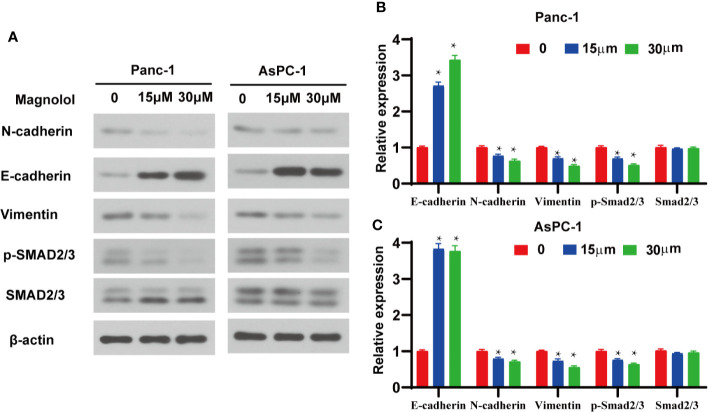
Magnolol inhibited epithelial-mesenchymal-transition (EMT) *via* the suppression of transforming growth factor-β1 (TGF-β1)/Smad Signalling. **(A)** Western blot analysis of protein levels of E-cadherin, N-cadherin, Vimentin, p-Smad2/3, Smad2/3 in Panc-1 and AsPC-1 cells treated with Magnolol(0, 15, 30 μM) for 48 h. **(B, C)** Histograms show the change of relative protein expression of E-cadherin, N-cadherin, Vimentin, p-Smad2/3, Smad2/3 in Panc-1 and AsPC-1 cells treated with Magnolol(0, 15, 30 μM) for 48 h. *P < 0.05.

TGF-β1 is the main inducer of EMT in the tumor microenvironment (17). To further explore the mechanism of action, the expression levels of EMT-associated genes and TGF-β signaling were detected in Magnolol-treated cells. Western blot showed elevated expression of E-cadherin and reduced expression of Vimentin in Magnolol-treated cells at the protein level (**Figures 5A–C**). Considering that the phosphorylation of Smads is a critical event in the TGF-β signaling, we determined the phosphorylation level of Smad2/3 in Magnolol-treated cells using western blot. The results showed that Magnolol reduced the phosphorylation of Smad2/3 in PANC-1 and AsPC-1 cells (**Figures 5A–C**).

### Magnolol Abolished TGF-β1-Induced Migration and Invasion *In Vitro*

**Figure 6:**
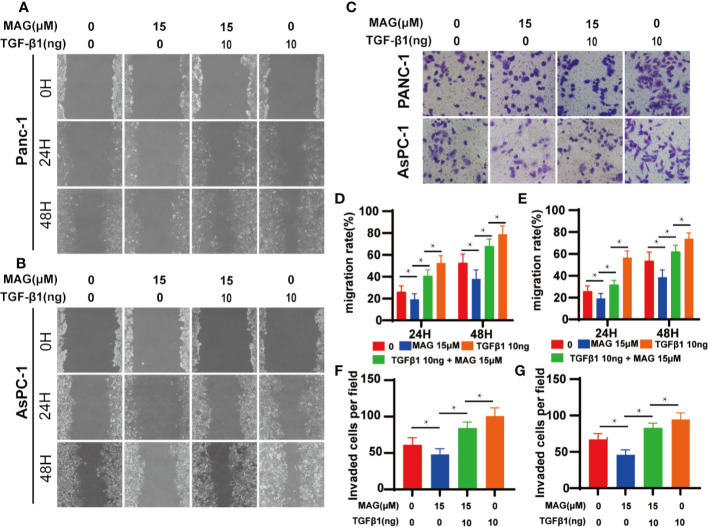
Magnolol abolished transforming growth factor-β1 (TGF-β1)-Induced Migration and Invasion *in vitro.* **(A, B)** The wound-healing of cells treated with with Magnolol or TGF-β1 was shown at 0, 24, and 48 h after scratching. **(D, E)** The migration rate of the scratch-wound assay was analyzed. **(E)** Morphological changes in Panc-1 and AsPC-1 cells after culture with Magnolol or TGF-β1. **(C, F, G)** Transwell invasion assay showed Magnolol treatment abolished TGF-β1 induced invasion of PANC-1 and AsPC-1 cells. *P < 0.05.

**Figure 7:**
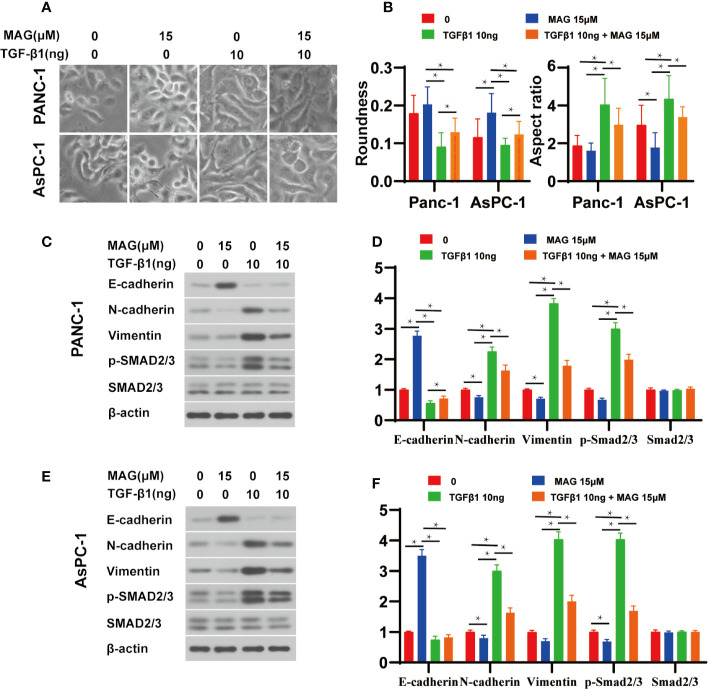
Magnolol abolished transforming growth factor-β1 (TGF-β1)-Induced epithelial-mesenchymal-transition (EMT) amd phosphorylation of Smad2/3 *in vitro*. **(A)** Morphological changes in Panc-1 and AsPC-1 cells after culture with Magnolol or TGF-β1. **(B)** Evaluation of cell morphology for Panc-1and AsPC-1 treated with Magnolol or TGF-β1. **(C, E)** Western blot results of E-cadherin, N-cadherin, Vimentin, p-Smad2/3, Smad2/3 in Panc-1or AsPC-1 treated with Magnolol or TGF-β1. **(D, F)** Histograms show the changes of relative expression of E-cadherin, N-cadherin, Vimentin, p-Smad2/3, Smad2/3 in Panc-1or AsPC-1 treated with Magnolol or TGF-β1. *P < 0.05.

Because Magnolol restrained the migration, invasion and altered the cell morphology, we further explored whether these effects of Magnolol relied on TGF-β1-induced EMT. Cancer cells were incubated with 10 ng/ml TGF-β1 cytokine alone or with 15 μM of Magnolol for 48 h. TGF-β1 significantly promoted the migration and invasion and Magnolol markedly impaired these effects of TGF-β1 in the PANC-1 and AsPC-1 cells (**Figure 6**). The morphological characteristics these cells were more mesenchymal after being induced by 10 ng/ml TGF-β1 alone than when incubated with 10 ng/ml TGF-β1 cytokine and 15 μM Magnolol (**Figure 7A**). The Roundness was significantly decreased while the Aspect ratio was significantly increased after being induced by 10 ng/ml TGF-β1 (**Figure 7B**). These effects were abolished by 15 μM Magnolol treatment (**Figure 7B**). Western blot showed that incubation of cancer cells with Magnolol and TGF-β1 simultaneously markedly decreased the levels of E-cadherin and phosphorylation of Smad2/3 compared with incubation with TGF-β1 alone (**Figures 7C–F**). These results demonstrated that Magnolol suppressed the aggressive behavior of these cells by inhibiting TGF-β1-induced EMT.

## Discussion

Current study was aimed to explore the therapeutic effect of Magnolol on pancreatic cancer *in vivo* and *in vitro*. Magnolol inhibited pancreatic tumor development in mouse model and suppressed the aggressive behavior of pancreatic cancer cells in a time- and concentration-dependent manner. As far as we know, the current study is the first to demonstrate these effects of Magnolol on pancreatic cancer. These data suggested that Magnolol inhibited TGF-β1-induced EMT *via* negatively regulating the phosphorylation of Smad2/3. Thus, we recommend Magnolol as a potential therapeutic for the treatment of pancreatic cancer. Further study will be needed to confirm molecular targets and loci.

Metastasis is a hallmark of tumors that contributes to cancer-associated death (18). The majority of patients are found with metastasis when diagnosed with pancreatic cancer (19). Tumor metastasis refers to a multi-step process of tumor cell migration from the primary site to distant sites (4). Epithelial-to-mesenchymal transition (EMT) features the evanishment of adhesion between cells accompanied by elevated levels of epithelial markers and reduced levels of mesenchymal markers (4). A series of pathways including Wnt, β-catenin, Notch, and tyrosine kinases is related to the induction of EMT. Among them, TGF-β1 is known as not only an inflammatory cytokine but also a pivotal inducer and sustainer of EMT (20). EMT is closely involved in not only invasion and metastasis but also proliferation and chemotherapy resistance. TGF-β-induced EMT plays an important role in PDAC progression, especially in metastases (20). Once bonded to its receptor on the cytomembrane, TGF-β induces phosphorylation of its downstream factors, including but not limited to SMAD proteins. The phosphorylated SMAD2/3 and SMAD4 subsequently form a regulatory complex that promotes the transcription of target genes (21).

Magnolol is a natural neolignan from magnolia plants and is reported to present antitumor, anti-oxidative, anti-inflammatory, and antibacterial effects (8). The antitumor properties of magnolol were reported to be related but not limited to the PI3K-AKT, p53, NF-κB, and p53-mediated mitochondrial pathways (9, 10, 22, 23). Unlike well-reported anti-proliferative effects, the anti-metastasis effects of magnolol are not as well studied. Magnolol has been reported to inhibit the ERK-modulated metastatic potential of hepatocellular carcinoma cells (24) and the HER2-modulated metastatic potential of ovarian cancer cells (25). Moreover, magnolol suppresses metastasis *via* matrix metalloproteinase-2/-9 activities in prostate carcinoma cells (26). However, the anti-metastatic function of magnolol remains unclear, especially in pancreatic cancer. In default of known research on this issue, we conducted this research.

The TGF-β/Smad signaling pathway is related to the occurrence of neurological disorders, fibrosis and malignancies (6). The TGF-β pathway presents a growth-repressive effect in the early stage of tumorigenesis in the pancreas by facilitating cell cycle arrest and apoptosis. Conversely, TGF-β signaling accelerates tumor progression in advanced-stage pancreatic cancer by inducing EMT, metastasis, invasion, migration, and immune evasion (27). In fact, the contribution of TGFβ to metastases has been well proven in pancreatic cancer (28, 29). Zhang et al. showed that Magnolol reduces bleomycin-induced rodent lung fibrosis by reducing TGF-β1 expression (30). Chei et al. reported that Magnolol does not influence apoptosis, but restrains cell migration, invasion and EMT in human colorectal tumor cells *via* suppressing the TGF-β pathway (31).

In the current study, we explored the effects of magnolol on pancreatic cancer *in vivo* and *in vitro*. First, the anti-tumor effect of magnolol was verified using orthotopic xenograft, and the changes in the expression of EMT markers were detected using immunohistochemical staining. Second, we studied the role of magnolol in impairing TGF-β1-mediated migration, invasion, proliferation and EMT in pancreatic cancer cells. Cancer cells were cultured with different concentrations of magnolol for 24, 48, and 72 h to explore the concentration-effect relationship. We chose concentrations of magnolol that suppress migration and invasion without inducing apparent cell proliferation arrest to perform our experiments. As published, 10 ng/ml of TGF-β1 induced EMT and facilitated pancreatic cancer cell migration and invasion (32, 33). We confirmed the EMT-inductive effects of that concentration in pancreatic cancer cells and then used it in our experiments. Our study verified that EMT played an important role in the aggressive actions of pancreatic cancer, while magnolol suppressed the migration, invasion, and proliferation of pancreatic cancer *via* EMT *in vivo* and *in vitro*.

There are also some limitations in our research. First, we studied the anti-tumor effects of magnolol on EMT by the Smad-dependent TGF-β pathway; however, the Smad-independent TGF-β pathway or other signaling pathways related to EMT and cancer were not detected. Second, while we found that magnolol suppressed phosphorylation of SMAD2/3, we did not verify the responsible molecular site of action. Third, we observed the tumor-suppressing effect of magnolol *in vitro*, and we focused on cancer cells to explore the antitumor effects of magnolol, but the effect of magnolol on the tumor environment also needs investigation. It will be necessary to perform in-depth research on the effects and mechanism of magnolol to determine whether magnolol could be used in the treatment of pancreatic cancer.

## Data Availability Statement

The raw data supporting the conclusions of this article will be made available by the authors, without undue reservation.

## Ethics Statement

The animal study was reviewed and approved by animal ethics committee of Xi’an Jiaotong University.

## Author Contributions

All authors listed have made a substantial, direct, and intellectual contribution to the work and approved it for publication.

## Funding

The project was supported by National Natural Science Foundation of China, NSFC (No:82003998, 81602401); Natural Science Foundation of Shaanxi Province (No: 2019JQ-969); and the Xi’an Jiaotong University Education Foundation, XJTUEF (No: xjj2018141).

## Conflict of Interest

The authors declare that the research was conducted in the absence of any commercial or financial relationships that could be construed as a potential conflict of interest.
